# Synthesis and Impedance Spectroscopy of Poly(*p*-phenylenediamine)/Montmorillonite Composites

**DOI:** 10.3390/polym13183132

**Published:** 2021-09-16

**Authors:** Udit Acharya, Patrycja Bober, Muhammed Arshad Thottappali, Zuzana Morávková, Magdalena Konefał, Jiří Pfleger

**Affiliations:** 1 Institute of Macromolecular Chemistry, Czech Academy of Sciences, 162 06 Prague, Czech Republic; acharya@imc.cas.cz (U.A.); bober@imc.cas.cz (P.B.); thottappali@imc.cas.cz (M.A.T.); moravkova@imc.cas.cz (Z.M.); magdalenakonefal@imc.cas.cz (M.K.); 2 Faculty of Mathematics and Physics, Charles University, 121 16 Prague, Czech Republic

**Keywords:** poly(*p*-phenylenediamine), montmorillonite, conductivity mechanism, impedance spectroscopy

## Abstract

Poly(*p*-phenylenediamine)/montmorillonite (PPDA/MMT) composites were prepared by the oxidative polymerization of monomers intercalated within the MMT gallery, using ammonium peroxydisulfate as an oxidant. The intercalation process was evidenced by X-ray powder diffraction. The FT-IR and Raman spectroscopies revealed that, depending on the initial ratio between monomers and MMT in the polymerization mixture, the polymer or mainly oligomers are created during polymerization. The DC conductivity of composites was found to be higher than the conductivity of pristine polymer, reaching the highest value of 10^−6^ S cm^−1^ for the optimal MMT amount used during polymerization. Impedance spectroscopy was performed over wide frequency and temperature ranges to study the charge transport mechanism. The data analyzed in the framework of conductivity formalism suggest different conduction mechanisms for high and low temperature regions.

## 1. Introduction

Composite materials based on polymers and inorganic materials have attracted increasing attention recently, thanks to the possibility of achieving superior properties by a combination of physical and chemical properties of the constituting components [1,2]. Such organic–inorganic composites are often applied as electrode materials in batteries [3,4] and supercapacitors [5]. Recently, composites composed of conjugated polymers or oligomers and clay fillers gained significant research interest due to their improved electrical, optical and mechanical properties, as well as corrosion resistance [6,7]. Montmorillonite (MMT) is a 2D layered phyllosilicate clay built of two tetrahedral and one octahedral sheet, which has been widely used in the preparation of nanocomposites due to its low cost, abundance in nature, zero toxicity, high specific surface area, swelling abilities and ion-exchange properties [8,9]. Various MMT composites with polyaniline [8,10], polypyrrole [11,12], poly (3,4-ethylenediaoxythiophene) [13] and polyphenylenediamine [14,15] were prepared by chemical [8,12] or electrochemical [13] polymerization and have been used as electrode material for oxygen reduction [16], anticorrosion coatings [6,11], sensors [17,18], conducting fillers [19], adsorbents [20] and supercapacitors [7].

In recent years, growing interest in the development of polyphenylenediamine composites can be observed [14,21,22,23,24,25]. Polyphenylenediamine is a conjugated polymer with a structure closely related to polyaniline; however, it possesses a higher nitrogen content and better solubility together with good redox properties, salt–base transition and environmental stability [23,26,27]. Nascimento et al. prepared poly(*p*-phenylenediamine)/MMT composites via oxidative polymerization and confirmed the successful formation of the polymer within the MMT gallery by extensive structural characterizations [14]. Ramya et al. studied the nonlinear optical properties of chitosan/*o*- or *p*-phenylenediamine/MMT composites by the Z-scan technique with picosecond and femtosecond laser pulses and revealed properties that make this material suitable for optical limiters [15]. Khelifa et al. examined the electrochemical properties of poly (*o*-phenylenediamie)/clay composites by cyclic voltammetry [28]. However, polyphenylenediamine/clay composites have still been described in the literature to a limited extent and impedance spectroscopy of them has not been yet reported. The conductivity of these composites can be contributed by electronic and ionic contribution, differing in their sensitivity to temperature, humidity and frequency response. Impedance spectroscopy is an important tool to elucidate the charge transport mechanism and the type of charge carriers in complex systems. For this, various formalisms are offered: conductivity, dielectric loss and modulus [29,30]. Knowing the conductivity mechanism is important for the development of the next generation of electronic devices, electrorheological fluids, as well as anticorrosive coatings, and optimizing their properties [31].

**Scheme 1:**
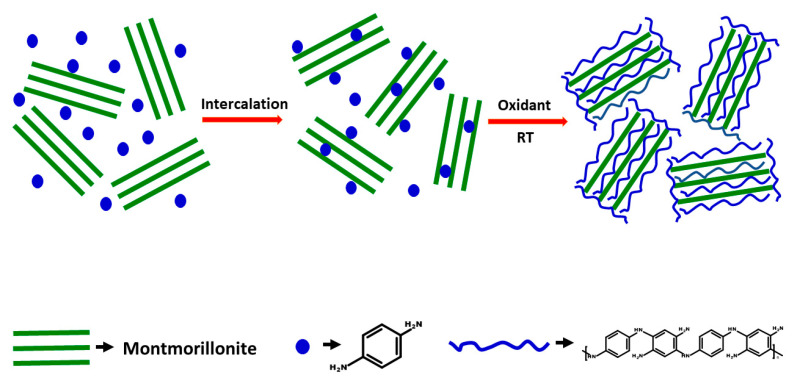
Schematic diagram of the preparation of PPDA/MMT composites.

In this work, a series of poly(*p*-phenylenediamine)/montmorillonite (PPDA/MMT) composites with various monomer to clay ratios has been prepared by in situ chemical oxidation of monomers within the MMT gallery (Scheme 1). Their structure and morphology were investigated by FT-IR and Raman spectroscopies, X-ray powder diffraction (XRD) and scanning electron microscopy (SEM). The impedance was measured in a wide frequency and temperature range and the AC conductivity mechanism was elucidated. We present here a detail impedance characterization to understand the conduction mechanism at different temperature regions. For the first time, the synergic effect in electrical, thermal, dielectric, impedance and modulus properties of PPDA/MMT composites has been investigated.

## 2. Materials and Methods

### 2.1. Preparation of Composites

*p*-Phenylenediamine dihydrochloride (≥98%) was purchased from Sigma-Aldrich (Buchs, Switzerland), ammonium peroxydisulfate from Lach:Ner (Neratovice, Czech Republic) and montmorillonite (Cloisite-Na^+^) from Southern Clay Products (Gonzales, TX, USA). All chemicals were used as received without any further purification.

In a typical process, 3.62 g of *p*-phenylenediamine dihydrochloride (PDA) was dissolved in 50 mL of deionized water. Various amounts of MMT (0 g, 2 g, 5 g, 10 g, 15 g, 25 g, 35 g and 50 g, respectively) were added into the PDA solution, and the mixture was sonicated for 5 h. The amount of 5.71 g of ammonium peroxydisulfate was dissolved in 50 mL deionized water and added to the PDA/MMT dispersion under magnetic stirring. The total concentration of monomer and oxidant in each polymerization mixture was 0.2 M and 0.25 M, respectively. After 1 h of intense stirring, the mixture was left undisturbed to polymerize for 24 h. The obtained precipitates were filtered and washed with deionized water and ethanol to remove by-products. The final products, poly(*p*-phenylenediamine)/montmorillonite (PPDA/MMT) composites with different amounts of MMT, were labeled as PPDA-X, where X accounts for the amount of MMT added to the reaction mixture.

### 2.2. Characterization

Morphology of composites was assessed using MAIA3 Tescan scanning electron microscope (Tescan, Brno, Czech Republic).

Thermogravimetric analysis (TGA) of the composites was performed on a Perkin Elmer Pyris 1 (Perkin Elmer, Waltham, MA, USA). Thermogravimetric Analyzer in a temperature range 35–800 °C at a rate of 10 °C min^−1^ with fixed air flow rate at 25 mL min^−1^.

FTIR spectra in the wavenumber range from 400 to 4000 cm^−1^ were obtained on the composites admixed in KBr pellets using a Thermo Nicolet NEXUS 870 FTIR spectrometer equipped with a DTGS detector. The instrument was purged with dry air. All spectra were corrected for the presence of water vapor and carbon dioxide in the optical path.

Raman spectra were measured with a Renishaw InVia Reflex Raman microspectrometer using 514 nm excitation provided by an Ar-ion laser. The scattered light was registered with a Peltier-cooled CCD detector (576 × 384 pixels) and analyzed by the spectrograph with holographic grating 2400 lines mm^−1^.

XRD measurements were performed using a pinhole camera (MolMet, Rigaku, Tokyo, Japan, upgraded by SAXSLAB/Xenocs) attached to a micro-focused X-ray beam generator (Rigaku MicroMax 003) operating at 0.6 mA and 50 kV. The camera was equipped with a vacuum version of the Pilatus 300K detector. The sample to detector distance, which was calibrated using a silver behenate powder sample, was 0.190 m. The scattering vector *q*, defined as *q* = 4π/λ·sin*Θ*, where λ is the wavelength and 2*Θ* is the scattering angle, covered the range 0.145–1.41 Å^−1^ (2*θ* = 2–20°).

The DC conductivity values were obtained on compressed pellets (diameter 13 mm and thickness 1.0 ± 0.2 mm) using van der Pauw method. A Keithley 230 Programmable Voltage Source in serial connection with a Keithley 196 System DMM was used as a current source. The potential difference was measured using a Keithley 617 Programmable electrometer or Keithley 181 nanovoltmeter, respectively, depending on the resistance of the sample. The DC conductivity measurements were carried out at stable ambient conditions at temperature 23 ± 1 °C and relative humidity 35 ± 5%. The frequency and temperature dependences of the impedance were measured in a quasi-steady-state regime using an Alpha-A Analyzer (Novocontrol Technologies, Montabaur, Germany) under applied AC voltage 1 V_rms_ in the frequency range 10^7^ to 10^−2^ Hz and temperature range 115 to 435 K with 20 K step in nitrogen atmosphere. For clarity, only curves for selected representative temperatures are shown in the figures. The pellets were placed in the sample holder between the gold-plated brass disk electrodes 13 mm in diameter.

## 3. Results and Discussion

**Figure 1:**
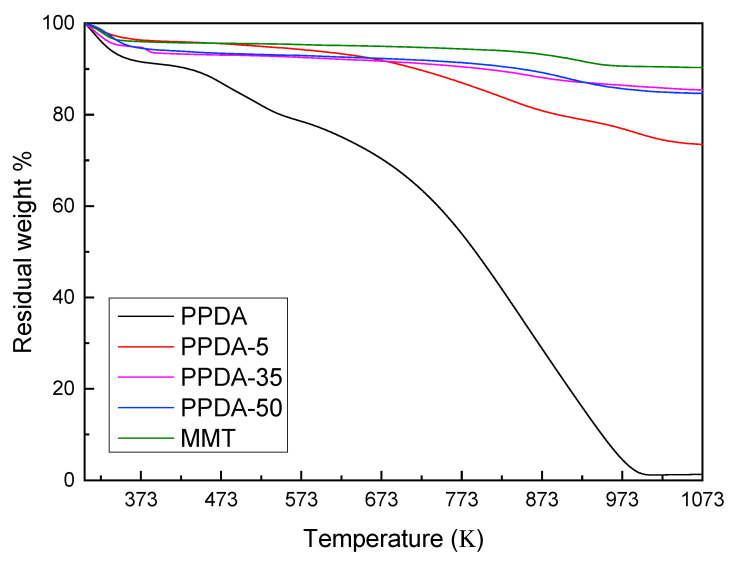
TGA curves of pristine MMT, PPDA and their composites.

In the first step of preparation, the *p*-phenylenediamine was intercalated into the MMT gallery, replacing Na^+^ cations of Na^+^-MMT with *p*-phenylenediamine cations. After the oxidative polymerization with APS, the amount of MMT in the resulting composites, listed in Table 1, was evaluated from TGA analysis (Figure 1). The results clearly demonstrate that even with a significant excess of monomers in the polymerization mixture, just around 7–8 wt% of PDA (Table 1) was intercalated into MMT and the rest remained in the surrounding aqueous medium and was removed later as a by-product during the purification procedure. This can be explained by the limited amount of PPDA that can be formed within the MMT gallery, based on limitations imposed by cation exchange in MMT. Additionally, when an excess of PDA was used, the MMT surface became coated with PPDA film, and the amount of PPDA in the composite reached 18–19 wt% (Table 1). These results are also in good agreement with the previously demonstrated synthesis of polyaniline/MMT composites [8]. The amount of MMT in the composites reached a plateau of ~93 wt% when less than ~25 wt% of PDA was used in the polymerization mixture; however, the properties and nature of such composites differ and are discussed below.

**Figure 2:**
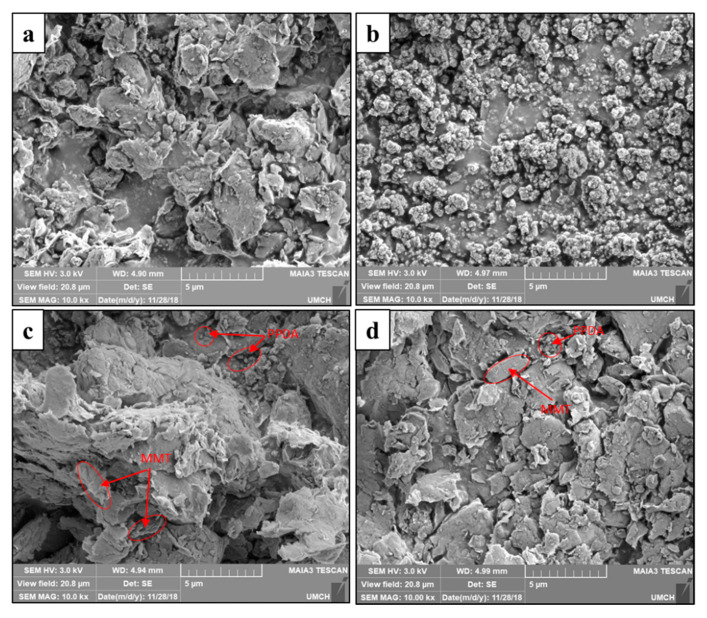
Scanning electron micrographs of pristine (**a**) MMT and (**b**) PPDA, and their composites prepared with various amounts of MMT: (**c**) PPDA-5 and (**d**) PPDA-50.

The morphology of both pristine components, MMT and PPDA, and their composites was investigated by SEM (Figure 2). Pristine MMT consists of flake-like particles of irregular shape (Figure 2a), while PPDA possesses globular morphology (Figure 2b), as already described in the literature [24]. After composite preparation, the morphology of MMT did not show considerable changes even at an excess of monomers (Figure 2c,d). No globular particles of PDPA were observed either. This confirms that the PPDA/MMT composites contain only PDPA embedded within the MMT galleries, or as a thin film covering MMT flakes, which cannot be seen in SEM. Free PPDA precipitate was evidently removed during the washing procedure.

The DC conductivity (Table 1) of all composites was found to be higher than for pristine PPDA (7 × 10^−11^ S cm^−1^), reaching ~10^−6^ S cm^−1^, the value close to pure MMT, refs.[32,33] for PPDA-35, where a higher amount of MMT was used in the polymerization mixture. 

### 3.1. X-ray Diffraction Analysis

**Figure 3:**
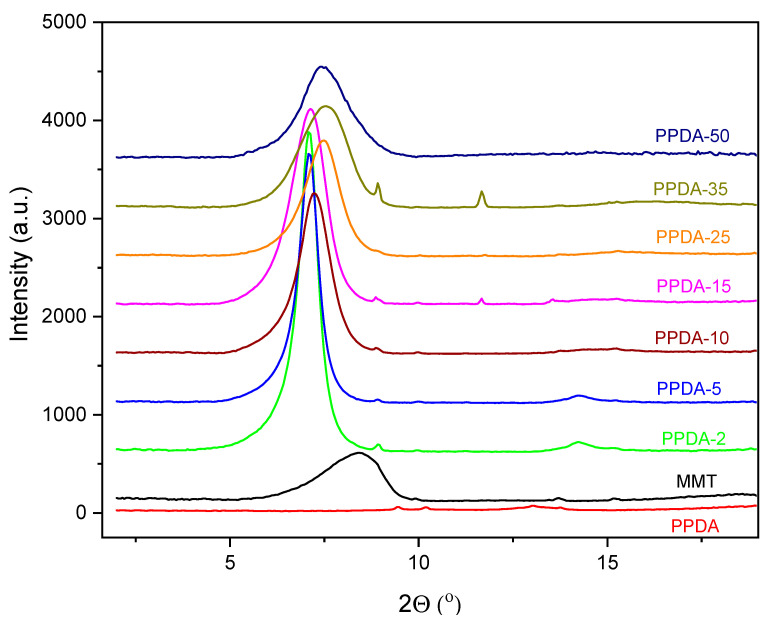
XRD patterns of pristine MMT, PPDA and their composites.

XRD patterns of pristine MMT and PPDA and their composites with different amounts of MMT are presented in Figure 3. For pristine MMT, a well-defined (001) Bragg reflection at 2θ = 8.41° was observed, corresponding to a basal spacing d of 10.5 Ǻ. For the composites, the reflection peaks of the (001) plane were displaced towards the lower 2θ values, which denotes the basal spacing expansion with respect to the pure MMT. The biggest change in basal spacing was observed for samples containing the lowest amount of MMT (PPDA-2 and PPDA-5), for which d = 12.5 Ǻ. The basal spacing expresses the sum of the thickness of one aluminosilicate layer and interlayer spacing, which can be affected by the size and orientation of interlayer ions. Assuming the thickness of the aluminosilicate layer of 9.8 Ǻ [34], the interlayer spacing for PPDA-2 and PPDA-5 composites expanded from 0.7 Ǻ (for pure MMT) to 2.7 Ǻ. This is a consequence of the intercalation process of PPDA chains in between the clay layers. The (001) reflections were in these cases sharp and narrow, indicating that the distribution of PPDA chains in the MMT galleries was uniform. A smaller increase in basal spacing was observed for samples containing a bigger amount of clay, reaching d = 11.7 Ǻ for the PPDA-50 sample. Moreover, the (001) diffraction peaks became much broader and less intense, which may suggest an inhomogeneous distribution of the polymer chains in the interlayers of MMT and possible partial exfoliation of MMT. These results suggest that the swelling of the MMT is related to the amount of PDA able to penetrate into MMT layers. A small signal around 2*θ* = 8.7°, visible in composite patterns, probably came from a small amount of MMT that was not intercalated during the synthesis of PPDA. The shift from 8.41° to 8.7° can be related to a decrease in basal spacing d, caused by the leaching of the interlayer cations [35]. The lack of this peak in the PPDA-50 pattern also suggests the possible partial exfoliation of MMT.

### 3.2. Infrared and Raman Spectroscopies

**Figure 4:**
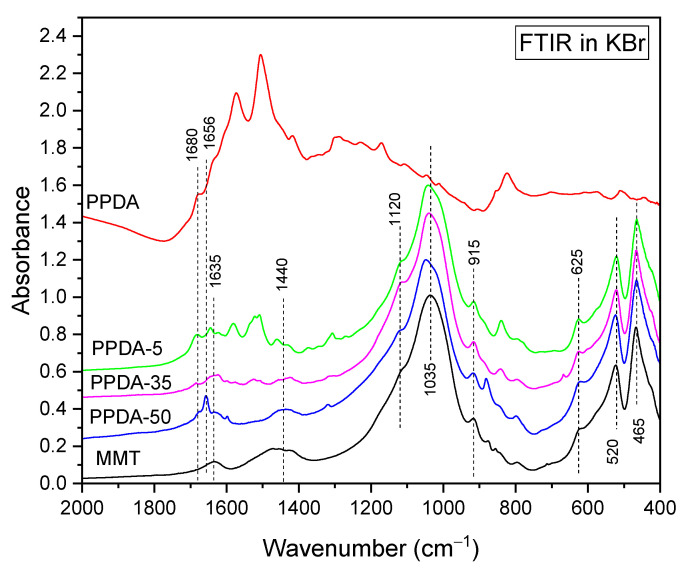
FTIR spectra of selected MMT, PPDA and their composites. The spectra were baseline corrected, normalized and shifted for clarity.

The FTIR spectra of the PPDA/MMT composites (Figure 4) mainly display the features of the MMT component: 1440 cm^−1^ (CO3 stretching of calcite impurity), 1035 cm^−1^ (Si–O stretching) with shoulders at 1120 cm^−1^ (Si–O stretching) and 915 cm^−1^ (Al–Al–OH bending), 520 cm^−1^ (Al–O–Si bending) with a shoulder at 625 cm^−1^, 465 cm^−1^ (Si–O–Si bending) and 1635 cm^−1^ (bending vibrations of water molecules hydrogen bonded to MMT or residual moisture in KBr) [36]. The bands associated with the PPDA component are well resolved only in the spectrum of the composite with the lowest amount of MMT (PPDA-5), and therefore the molecular structure of the PDA oxidation products will be discussed in detail based on Raman spectroscopy. However, let us mention the peak at 1656 cm^−1^ in the spectrum of PPDA-50. We suspect this band can be related to C=O stretching of the benzoquinone structure [37,38,39].

**Figure 5:**
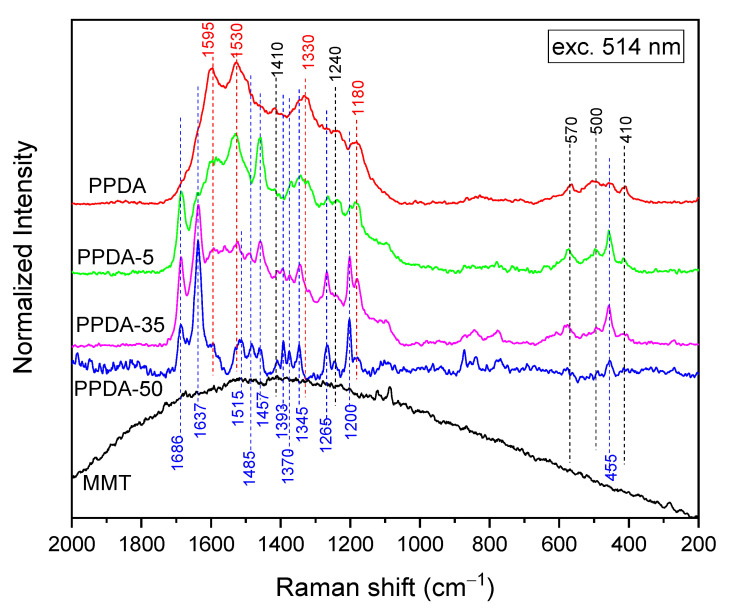
Raman spectra of selected MMT, PPDA and their composites excited with the 514 nm laser line. The spectra were baseline corrected, normalized and shifted for clarity.

For the Raman spectroscopy (Figure 5), excitation at 514 nm was chosen to minimize the background caused by MMT fluorescence. The spectrum of the neat PPDA resembles the PANI-like structure reported many times in previous literature. The main bands are observed at 1595 cm^−1^ (C=C stretching in a quinonoid ring), 1530 cm^−1^ (N–H stretching), 1330 cm^−1^ (C~N^+●^ stretching vibration related to a delocalized polaron structure), 1180 cm^−1^ (C–H deformation), 570 cm^−1^, 500 cm^−1^, 455 cm^−1^ and 410 cm^−1^ (ring deformations) [8,23,40,41]. The spectra of all composites mainly show peaks related to MMT, but there are also some peaks, visible particularly in the PPDA-50 composite with the highest amount of MMT, which occur neither in PPDA nor in MMT. Namely, the relatively strong and sharp peak at 1686 cm^−1^ observed for the PPDA-50 composite has not yet been observed for any PANI of PPDA-related material. This band can be related to the NH_2_ end-groups [42]. The strong peak at 1637 cm^−1^ dominates the spectrum. It was formerly assigned to ring stretching in phenazine-like [43] or oxazine-like structures [44]; however, in detailed spectroscopy studies of phenazine structures [43,45], this peak was only observed in neutral N-phenyl phenazine. However, due to the expected high local acidity in the MMT interlayer area, N-phenyl phenazine should occur there only in protonated form. The strong peak at 1637 cm^−1^ could also originate in NH deformation vibrations [46] or benzenoid ring stretching [47,48], which seems to be a more likely assignment. The band at 1515 cm^−1^ is assigned to the N–H deformation of the PANI-like structure [49,50,51,52]. The bands at 1485 and 1457 cm^−1^ belong to C=N stretching in quinonoid units [53], and N=N stretching in an azobenzene structure can contribute to the latter [54]. The bands at 1393, 1370 and 1345 cm^−1^ can be attributed to C~N^+●^ stretching in polaron structures with varying delocalization lengths [40,55]. The bands at 1265 and 1200 cm^−1^ belong to C–H deformation in quinonoid and benzenoid rings, respectively. The band at 455 cm^−1^ is due to out-of-plane ring deformations [40]. In conclusion, a generally PANI-like structure was observed in the PPDA-50 composite, but given the strong signal of NH_2_ groups and the contribution of highly localized polarons, the material intercalated in MMT seems to be of oligomer nature. The Raman spectra of PPDA-35 and PPDA-5 composites reflect both features of the oligomer observed in PPDA-50 and of the PPDA polymer, with the intensity of the above discussed peaks at 1393, 1370, 1686 and 1637 cm^−1^ increasing with the MMT content.

### 3.3. Impedance Spectroscopy

**Figure 6:**
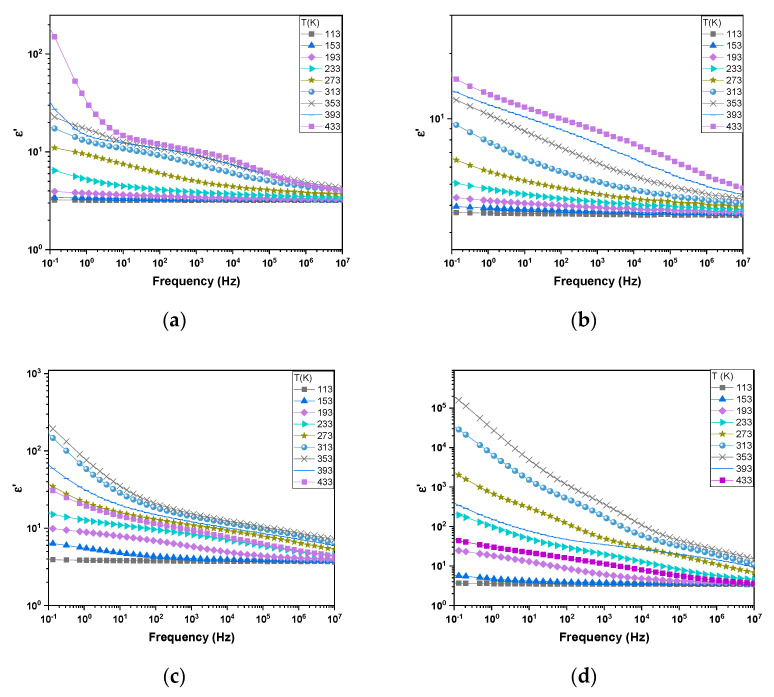
Variations in real part of permittivity with frequency at different temperatures for (**a**) PPDA and PPDA/MMT composites: (**b**) PPDA-5, (**c**) PPDA-35 and (**d**) PPDA-50.

**Figure 7:**
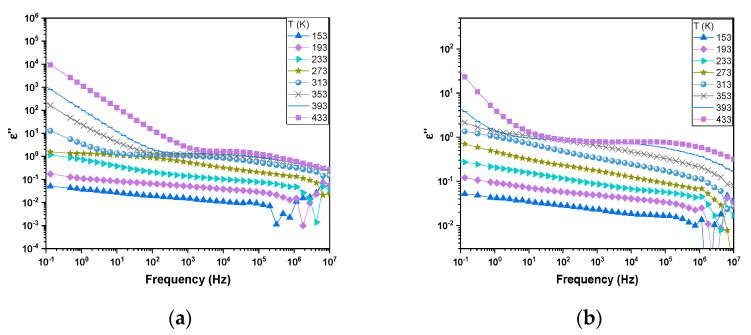

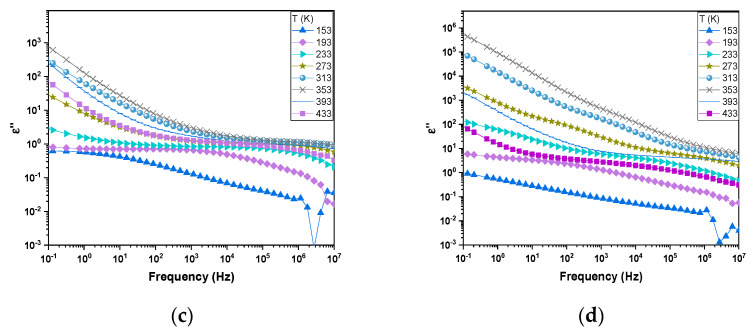
Variations in imaginary part of permittivity with frequency at different temperatures for (**a**) PPDA and PPDA/MMT composites: (**b**) PPDA-5, (**c**) PPDA-35 and (**d**) PPDA-50.

The real part (*ε*′) and imaginary part (*ε*″) of complex permittivity ε* (ε* = *ε*′ − *jε*″) measured as a function of frequency (10^−2^ Hz to 10^7^ Hz) at different temperatures (113 K ≤ T ≤ 433 K) are shown in Figure 6 and Figure 7. When interpreting these data, we should consider conductivity being contributed by both the electronic and ionic parts. At lower frequencies, an increase in the dielectric constant with decreasing frequency is typical for electrode polarization due to the presence of mobile ions. In lower frequency regions, different values of slopes in *ε*″ vs. f curves imply ion transport if slope~ −1, whereas slope > −1 means the polarization effect. The decreasing value of slope at a higher frequency represents the caged movement of ions, i.e., ion–ion interactions and short time regime correlations [56]. The Maxwell–Wagner model can be utilized to understand the dielectric response of these materials [57]. These polarizations are also temperature-dependent, as shown in Figure 7. As the temperature increases, ion dissociation takes place, which ultimately results in an increase in dielectric polarization. Moreover, interfacial polarization also occurs because of the buildup of charges at the grain boundaries. At a lower temperature, dissociated ions are not available, and a small value of permittivity is assigned to polarons dominant in PPDA, which are almost in frozen condition. With the gradual increase in temperature, these polarons inside grains separated by grain boundaries result in the Maxwell–Wagner effect at lower frequencies. In the case of PPDA-5, the value of *ε*′ and *ε*″ is small due to the smaller content of MMT, which is directly proportional to water content since water molecules are captured in between MMT layers and form ionic dipoles [57]. This is also evidenced in Figure 6c,d, where the *ε*′ value decreases after exceeding 353 K since water starts to evaporate [58]. The variation in the dissipation factor (tan *δ* = *ε*″/*ε*′) with temperature at different frequencies decreases with increasing frequency at all temperatures, which is in a good agreement with Koop’s phenomenological model [59]. The composites PPDA-35 and PPDA-50 attain a low value of tan *δ*, which is due to the partial exfoliation of the MMT gallery as evidenced by XRD.

**Figure 8:**
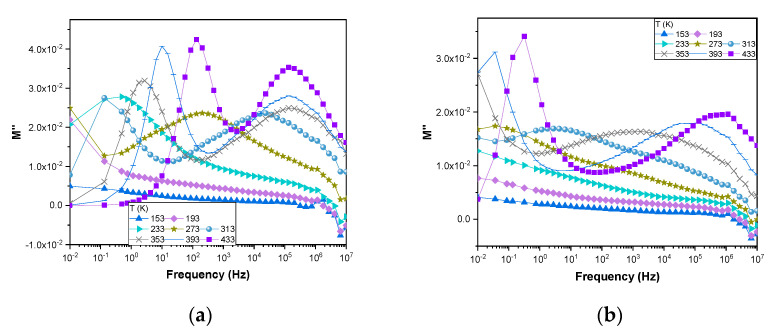

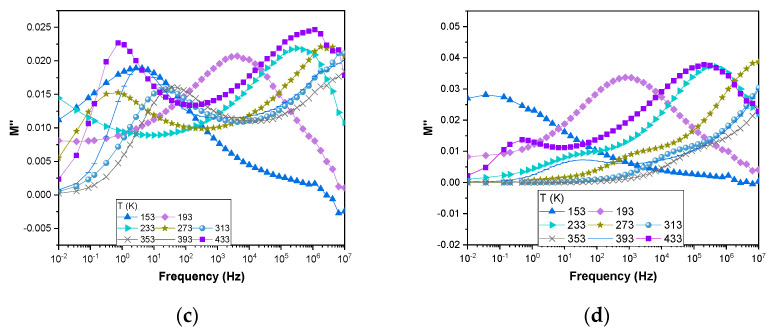
Variations in imaginary part of modulus with frequency with temperature for (**a**) PPDA and PPDA/MMT composites: (**b**) PPDA-5, (**c**) PPDA-35 and (**d**) PPDA-50.

To better discuss relaxation processes of the impedance spectra and suppress the electrode polarization effects, the complex dielectric modulus was obtained from the real and imaginary part of the permittivity.

(1) $$M^{*}=\frac{1}{\varepsilon^{*}}=\frac{1}{\left(\varepsilon -j\varepsilon^{\prime \prime }\right)}=M^{\prime }+jM^{\prime \prime }=\frac{\varepsilon^{\prime }}{{\left(\varepsilon^{\prime }\right)}^{2}+{\left(\varepsilon^{\prime \prime }\right)}^{2}}+j\frac{\varepsilon^{\prime \prime }}{{\left(\varepsilon^{\prime }\right)}^{2}+{\left(\varepsilon^{\prime \prime }\right)}^{2}}$$

where *M′* and *M″* are the real part and imaginary part of the complex dielectric modulus (M*), respectively [60]. Figure 8 shows the frequency dependence of the imaginary part of the modulus at different temperatures. In fact, the low frequency peak corresponds to the long-range migration of ions, whereas the high frequency peak represents spatially confined ions within potential wells and localized short distance motion within the domains [56].

**Figure 9:**
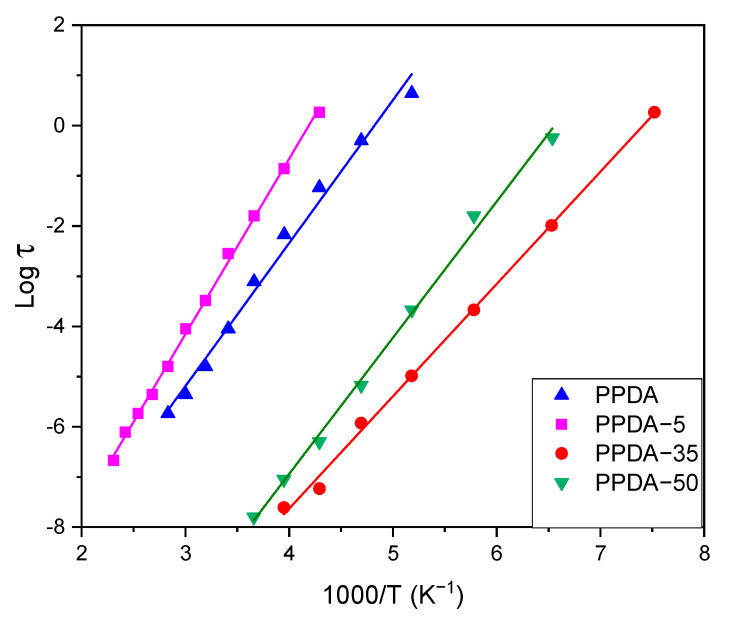
Plot of relaxation time vs. reciprocal temperature for PPDA and PPDA/MMT composites.

The values of relaxation time $\tau =\frac{1}{w_{max}}$ corresponding to the frequency of the low-frequency maximum *f_max_* of the *M″* frequency spectrum are plotted in Figure 9 as a function of temperature. This follows an Arrhenius law with the activation energy *Ea* being 0.56, 0.69, 0.44 and 0.53 eV for pristine poly(*p*-phenylenediamine), PPDA-5, PPDA-35 and PPDA-50, respectively.

**Figure 10:**
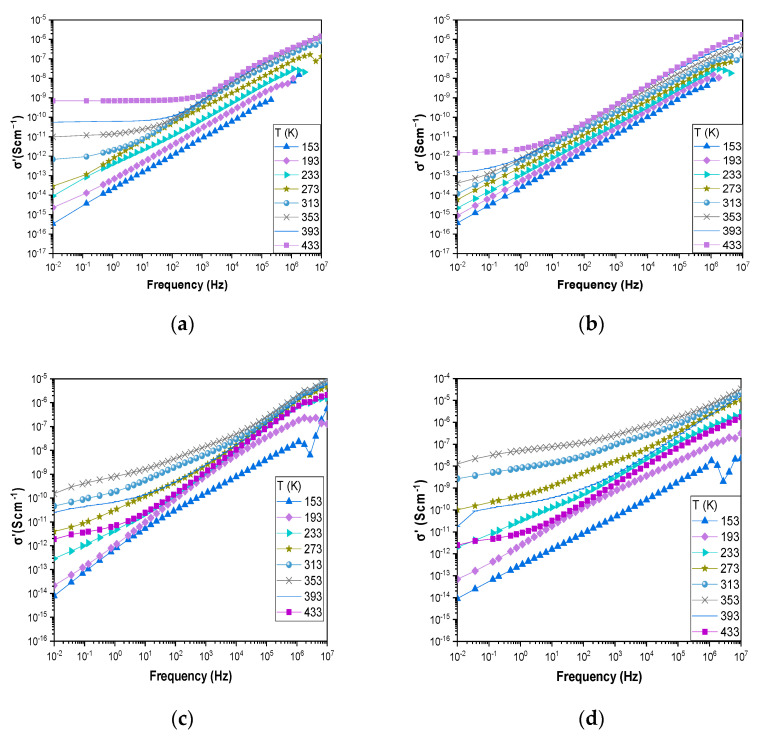
Variations in real part of AC conductivity with frequency at different temperatures for (**a**) PPDA and PPDA/MMT composites: (**b**) PPDA-5, (**c**) PPDA-35 and (**d**) PPDA-50.

**Figure 11:**
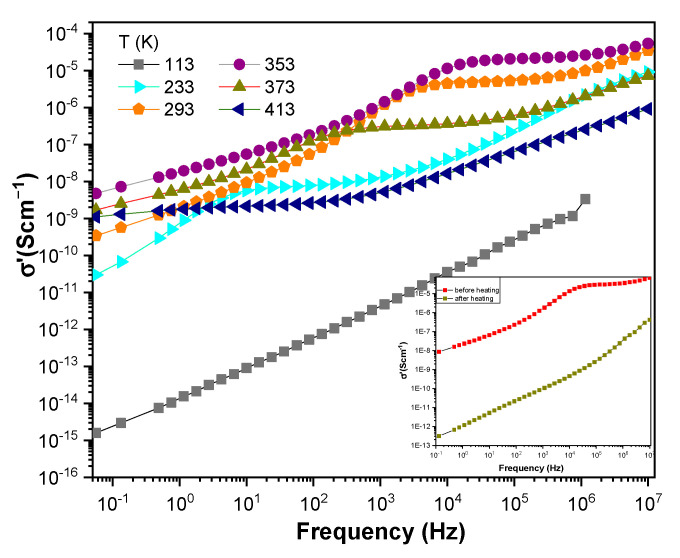
Variations in real part of AC conductivity with frequency at different temperatures for the pure montmorillonite. Inset shows the variation in conductivity at 293 K before and after heating to 433 K.

**Figure 12:**
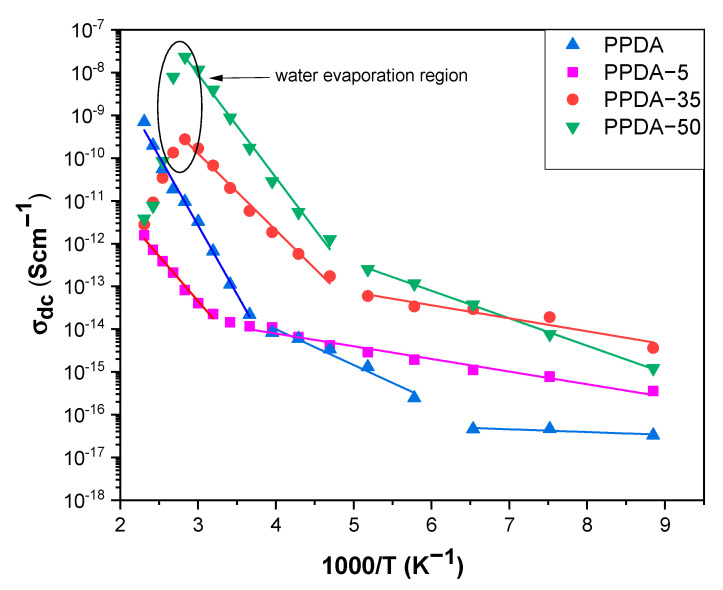
Variation in DC conductivity with reciprocal temperature. The solid lines represent fitting according to Arrhenius equation.

The frequency dependences of the real part of conductivity at different temperatures are shown in Figure 10 for the polymer and composites and Figure 11 for montmorillonite. The real part of the conductivity obeys a power law known as Jonscher’s law given by Equation (2) within the considered frequency and temperature ranges:

(2) $$\sigma^{\prime }=\sigma_{dc}+Aw^{s}$$

where *σ_dc_* is the DC conductivity obtained by extrapolating *σ′* to the low frequency limit. The term *Aw^s^* is the dispersive component of AC conductivity, which represents the degree of interaction between mobile ions and the lattices. Here, *A* is the pre-exponential factor describing the strength of the polarizability, and *s*, which is a power law exponent, within 0 < *s* < 1, is a measure of the correlation degree. The behavior of s on temperature provides an insight into the conduction mechanism. The power law dependences point to inhomogeneity in the composites and that the ion transport plays the crucial role. At room temperature and lower frequencies, the composites PPDA-35 and PPDA-50 show a higher value of conductivity than pristine materials. The same is observed in the case of DC conductivity measured with the four-probe van der Pauw method (Table 1). This increase in conductivity is related to the intercalation of PPDA chains between the layers of MMT, which leads to higher ordering in polymer chains causing more a compact structure compared to pristine PPDA. The higher DC conductivity values obtained from the DC four-probe method compared to impedance spectroscopy can be explained by the elimination of the contact resistance in the former case. Figure 12 shows the Arrhenius plots of *σ_dc_* against 1/*T* for all the samples according to Equation (3):

(3) $$\sigma_{dc}=\sigma_{0}\;\exp\;(-\frac{E_{dc}}{k_{B}T})$$

The calculated values of activation energy (*E_dc_*) are listed in Table 1. It is seen that different activation energies exist at high and low temperatures, which indicates the dissociation of ions with an increase in temperature, hence increasing the conductivity at high temperatures, but at a low temperature, the ions do not have enough energy to hop to neighboring sites and remain caged. For the sample PPDA-35 and PPDA-50, the change in the slope of the curve occurs at 200 K, which is attributed to the motion of the water–polymer complex in the amorphous region [61], whereas for pristine PPDA and PPDA-5 it is at 273 K, revealing the existence of phase transition that might be associated with alternation in local structures. The lowest activation energy is found for the sample PPDA-35, which means less energy is required to migrate the ions from the binding coordinating site to another site. The decrease in conductivity for PPDA-35 and PPDA-50 above 353 K is again associated with the evaporation of intercalated water. For the low temperature region where the mobile charge carriers are mainly polarons but not ions, the activation energies are sufficiently low and following the opposite trend compared to that in the higher temperature region. Pristine PPDA has the lowest and the composites with more MMT content have higher values of activation energy since the movements of polarons inherited in the polymer are hindered with the addition of MMT, and DC conductivity is thus not only a function of temperature as expressed by the Arrhenius equation [62]. Additionally, pristine PPDA shows intermediate activation energy, which is attributed to the contribution of polymer segmental motion.

**Figure 13:**
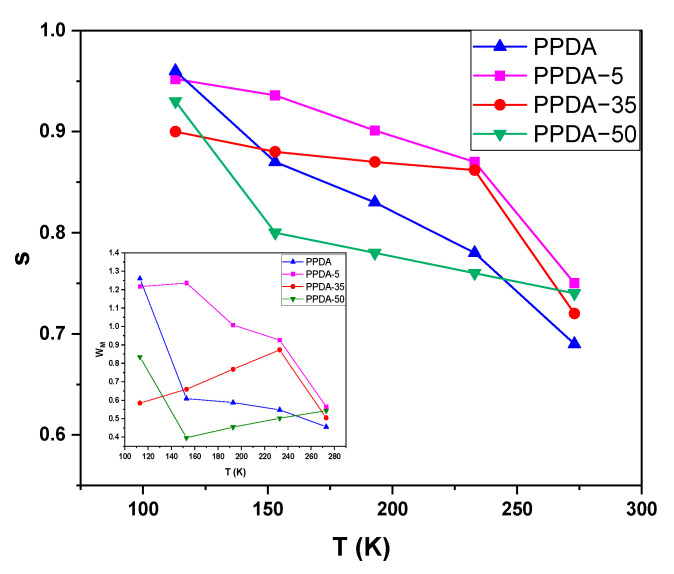
Variation in exponent parameter s with temperature for PPDA and PPDA/MMT composites. The solid lines are drawn for clarity. Inset shows the variation in *W_M_* with temperature.

From Figure 12, it is clearly seen that two different activation energies exist at different temperature regions. This suggests that two conduction mechanisms prevail, one at lower temperature (polaronic) and another at high temperature (ionic). The temperature dependences of *s* values obtained from the linear fit of the *σ_ac_* frequency dependencies at the linear region are plotted in Figure 13 for the lower temperature region. To study the conduction mechanism at lower temperatures, various models have been proposed in the literature to explain the predominant conduction mechanisms according to the shape of the temperature dependence of exponent *s*. For the case of *s* increasing with increasing temperature, a small polaron conduction mechanism has been suggested, whereas if *s* decreases with increasing temperature then the mechanism is called correlated barrier hopping. If *s* is independent with temperature, then quantum mechanical tunneling is expected [63]. In our case, the exponent *s* decreases as the temperature increases, which is attributed to correlated barrier hopping (CBH) of polarons. The frequency dependence according to the CBH model can be expressed by Equation (2), with s given by Equation (4) [64].

(4) s=1−6kBT[WM−kBTln(1ωτ0)]

where *W_M_* is the polaron binding energy, *τ*_0_ is the characteristic relaxation time and *k_B_* is the Boltzmann’s constant. For large *W_M_*/*K_B_T*, Equation (4) can be deduced as:

(5) $$W_{M}=\frac{6k_{B}T}{1-s}$$

The values of *W_M_* calculated at the low temperature region are shown in the inset of Figure 13 using the experimental values of *s*. The values obtained indicate that single polarons are the major charge carriers but not bipolarons since for bipolaronic transport values of *W_M_* must be almost four times higher [64]. It is seen that at the lowest measured temperature, these single polarons have a higher binding energy for pristine PPDA and PPDA-5 than in the composites PPDA-35 and PPDA-50, but after the gradual increase in temperature similar values are attained.

As seen in Figure 11, the dependence of the real part of conductivity shows the plateau that allows the determination of DC conductivity of ~10^−6^ S/cm, which is in a good agreement with values reported in the literature [32,33]. The conductivity decreases above 353 K due to the evaporation of intercalated water. This is similar to the behavior described above for the composites with a higher content of MMT. The conductivity decreases drastically after heating to 433 K, referring to the humidity dependence of montmorillonite and ions playing a dominant role in the conduction mechanism [65,66].

## 4. Conclusions

PPDA/MMT composites were successfully synthesized by a two-step procedure, viz., the intercalation of monomers, followed by oxidative polymerization. Even though, contrary to the initial expectations, the introduction of various amounts of MMT into the polymerization mixture led to similar amounts of the MMT in obtained composites, the variation in the initial monomer-to-MMT ratio allowed us to obtain series of composites with diverse conductivity. Spectroscopy characterization revealed that such composites differ in the polymeric or oligomeric nature of the organic part. Both AC and DC conductivities of composites were found to be higher than the corresponding polymer. Impedance spectroscopy studied over a wide range of frequency and temperature points to the change in the conduction mechanism from polaronic conduction arising from the polymer in low temperatures to ionic conduction in higher temperatures inherited in MMT. Such composites can be applied as anticorrosion coatings, sensors, electromagnetic shielding or in electrorheology.

## Figures and Tables

**Table 1:** The montmorillonite content and DC conductivity of PPDA/MMT composites.

Sample Code	PDA Content in Polymerization Mixture, wt%	MMT Content in Polymerization Mixture, wt%	PPDA Content in Composites, wt%	MMT Content in Composites, wt%	Conductivity, S cm^−1^ (Measured by Van der Pauw Method)	Activation Energy (Ea) (eV)		
						Evaluated from M″ vs. f	Evaluated from DC Conductivity vs. 1/T	
							High Temperature Region	Low Temperature Region
PPDA	100	0	100	0	7 × 10^−11^	0.57	0.64	0.02
PPDA-2	64	36	19	81	3 × 10^−11^	-	-	-
PPDA-5	42	58	18	82	1 × 10^−11^	0.69	0.58	0.05
PPDA-10	26	74	8	92	2.5 × 10^−8^	-	-	-
PPDA-15	19	81	8	92	3.4 × 10^−8^	-	-	-
PPDA-25	13	87	7	93	2.2 × 10^−7^	-	-	-
PPDA-35	9	91	6	94	1 × 10^−6^	0.44	0.34	0.06
PPDA-50	7	93	7	93	9.2 × 10^−7^	0.54	0.42	0.12

## Data Availability

We have no depository of publicly archived datasets analyzed or generated during the study. Data are available on request; please contact authors on their e-mail addresses.
